# Enhanceosome transcription factors preferentially dimerize with high mobility group proteins

**DOI:** 10.1186/s12918-016-0258-3

**Published:** 2016-02-04

**Authors:** Aleksander Jankowski, Paulina Obara, Utsav Mathur, Jerzy Tiuryn

**Affiliations:** Faculty of Mathematics, Informatics and Mechanics, University of Warsaw, Banacha 2, Warsaw, 02-097 Poland; Faculty of Mathematics and Computer Science, Jagiellonian University, Łojasiewicza 6, Kraków, 30-348 Poland; Current address: Genome Biology Unit, European Molecular Biology Laboratory, Meyerhofstraße 1, Heidelberg, 69117 Germany

**Keywords:** Enhanceosome, Transcription factors, Dimerization, High mobility group genes, Enhancers

## Abstract

**Background:**

The enhanceosome is an enhancer located upstream of the human interferon *β* gene, bound by transcription factor (TF) complex of extremely rigid structure. Within these rigid constraints, even a slight change of distances between transcription factor binding sites (TFBS) results in loss of functionality of the enhanceosome. We hypothesized that smaller subunits of the enhanceosome may entail TF complex formation in other regulatory regions.

**Results:**

In order to verify this hypothesis we systematically searched for dimerization preferences of the TFs that have TFBS in the enhanceosome. For this we utilized our recently developed tool, TACO. We performed this computational experiment in a cell-type–specific manner by utilizing cell-type–specific DNase-seq data for 105 human cell types. We also used 20 TRANSFAC motifs comprising not only the usual TFs constituting the enhanceosome but also the architectural proteins of High Mobility Group I(Y) (HMG I). A similar experiment used 42 DNase-seq data sets for mouse cell types. We found 137 statistically significant dimer predictions in the human genome, and 37 predictions in the mouse genome, that matched the positioning on the enhanceosome with ±2 bp tolerance. To characterize these predicted TF dimers, we performed functional analysis (Gene Ontology enrichment) for sets of genes which were in the neighbourhood of predicted dimer instances. A notable feature of these instances is that (1) most of them are located in introns of genes, (2) they are enriched in regulatory states, and (3) those instances that are located near transcription start sites are enriched for inclusion in computationally predicted enhancers. We also investigated similarity of dimer predictions between human and mouse.

**Conclusions:**

It follows from our experiments that, except for homodimer formed by IRF proteins, the rest of the dimers were formed exclusively between one of the transcriptional activators (ATF-2/c-Jun and IRF) and a HMG I protein. NF- *κ*B did not participate in forming dimers with other proteins. Dimers predicted in mouse were fully contained in those predicted in human, with exactly the same spacing and orientation. Intriguingly, in most of the cases the enhanceosome motifs have 1 bp wider spacing than the corresponding dimers predicted genome-wide, which is likely caused by the overall 3D structure constraints of the enhanceosome-bound complex.

**Electronic supplementary material:**

The online version of this article (doi:10.1186/s12918-016-0258-3) contains supplementary material, which is available to authorized users.

## Background

Transcriptional activation of the human interferon *β* (IFN- *β*) gene is governed by an enhancer, termed the *enhanceosome*. This enhancer is located in the human genome on chromosome 9 and forms a region of length 57 bp, which is 44 bp upstream of IFN- *β*. The enhanceosome is one of the best studied enhancers [1]. It is an excellent example of combinatorial interaction between distinct regulatory elements. This enhancer is bound by NF- *κ*B (p50/RelA), ATF-2/c-Jun and interferon regulatory factor (IRF) proteins. The first two proteins are heterodimers. All together these transcriptional activators have well characterized eight binding sites in the enhanceosome (the heterodimers occupy two binding sites, each, and IRFs occupy four binding sites); see [1] for a review. NF- *κ*B and ATF-2/c-Jun proteins recognize two positive regulatory domains (PRDs): PRD II and PRD IV, respectively, while IRFs recognize the other two domains: PRD I and PRD III. Notably, many neighbouring binding sites in the enhanceosome overlap.

The process of activation of interferon *β* is quite intricate and highly ordered as described for example in [2]. Since 2007 high resolution crystal structures that concern portions of the enhanceosome with portions of relevant transcription factors are known [3–5]. As a result of molecular dynamics simulations of different motif combinations of the enhanceosome it was argued [6] that the specificity of the enhancer is attained in this case via cooperative binding of the neighbouring partners.

It was proposed by Arnosti and Kulkarni [7] that enhancers exhibit a wide spectrum of adopted constraints put on the structure of their transcription factor (TF) binding sites, ranging from those with a very flexible structure, termed billboards, and ending up in enhancers with a very rigid structure, whose primary example is the enhanceosome. Rigidity of the enhanceosome shows up when one changes the arrangement of the transcription factor binding sites (TFBS) of the involved transcriptional activators of IFN- *β*: even slight change in distance or orientation of TFBS results in a dramatic drop of the activity of gene expression [2].

In addition to the above mentioned transcriptional activators, an important role in fine tuning the specificity in gene expression is played by architectural proteins of the mammalian high mobility group HMG I(Y) [8]. HMG I and HMG Y are alternative RNA splicing variants encoded by the same gene. It is known that the role of HMG I(Y) proteins in the first step of enhanceosome assembly is the recruitment of NF- *κ*B and ATF-2/c-Jun by allosteric changes induced in the DNA [8]. However, in the second step, upon completion of the enhanceosome assembly process, it is required that HMG I(Y) proteins form protein-protein interactions with the activators. HMG I(Y) proteins have three binding sites in positive regulatory domains of the enhanceosome: two in PRD IV, and one in PRD II, which we refer to as HMG-A, HMG-B and HMG-C, respectively.

The functional role of the involved TFs on the level of expression of IFN- *β* is not precisely determined yet. For example it has been recently reported [9] that the role of these TFs is not so symmetrical as one might expect. The authors report that the role of IRFs is critical for the expression of INF- *β* when performing an experiment in a transgenic mouse, but it is not so with the NF- *κ*B subunits (p50 and RelA). In another work in the same vein [10] it has been reported that the IFN- *β* enhanceosome region is not sufficient for maximal gene induction in response to stimulation with bacterial lipopolysaccharide (LPS). This study also identified a cluster of NF- *κ*B sites in the 3’ downstream region of the gene.

Given the above remarks, we set out a hypothesis that smaller subunits of the enhanceosome may entail TF complex formation in other regulatory regions. More specifically we want to investigate which dimers (pairs of proteins) involved in enhanceosome assembly (transcriptional activators and architectural proteins) are preferably used in other regulatory elements.

In order to address these questions we applied TACO [11], our recently developed tool to predict TF dimers from DNA sequence by incorporating position weight matrices (PWMs) and information about regions of open chromatin. The selection of statistically overrepresented dimer structures (as defined by a pair of PWMs, offset between TFBS, and their mutual orientation) is done in a cell-type–specific manner, as described earlier [12]. Here, we consider 20 TRANSFAC motifs [13] that represent transcriptional activators and HMG I(Y) binding affinities with hits in the enhanceosome. We also used in this experiment DNase-seq data for 105 human cell types. We found 137 dimer predictions that matched the positioning on the enhanceosome with ±2 bp tolerance.

Analysis of the above mentioned computational experiment suggests the following key observations: (1) among transcriptional activators only the IRF proteins form a homodimer that is utilized on a genome-wide scale, (2) all other predicted dimers involve HMG I(Y) proteins and ATF-2/c-Jun or IRF transcription factors, (3) the majority of dimer instances is located within introns of genes.

We also repeated the same experiment with mouse genome and mouse DNase-seq data for 42 cell types. In this case, 37 predictions matched those present in the mouse enhanceosome with ±2 bp tolerance. The key observations for the mouse enhanceosome are similar to the above observations for human. A noticeable relation linking predictions in human and in mouse is that even though the latter form a subset of the former, every predicted dimer structure in mouse is also predicted in human with exactly the same arrangement. It is also noteworthy that genes that contain predicted dimer instances in human and in mouse have similar GO term annotations (*p*-value <1.0*e*−7, Fisher’s exact test).

## Results

### Predicted dimers in various cell types

**Fig. 1:**
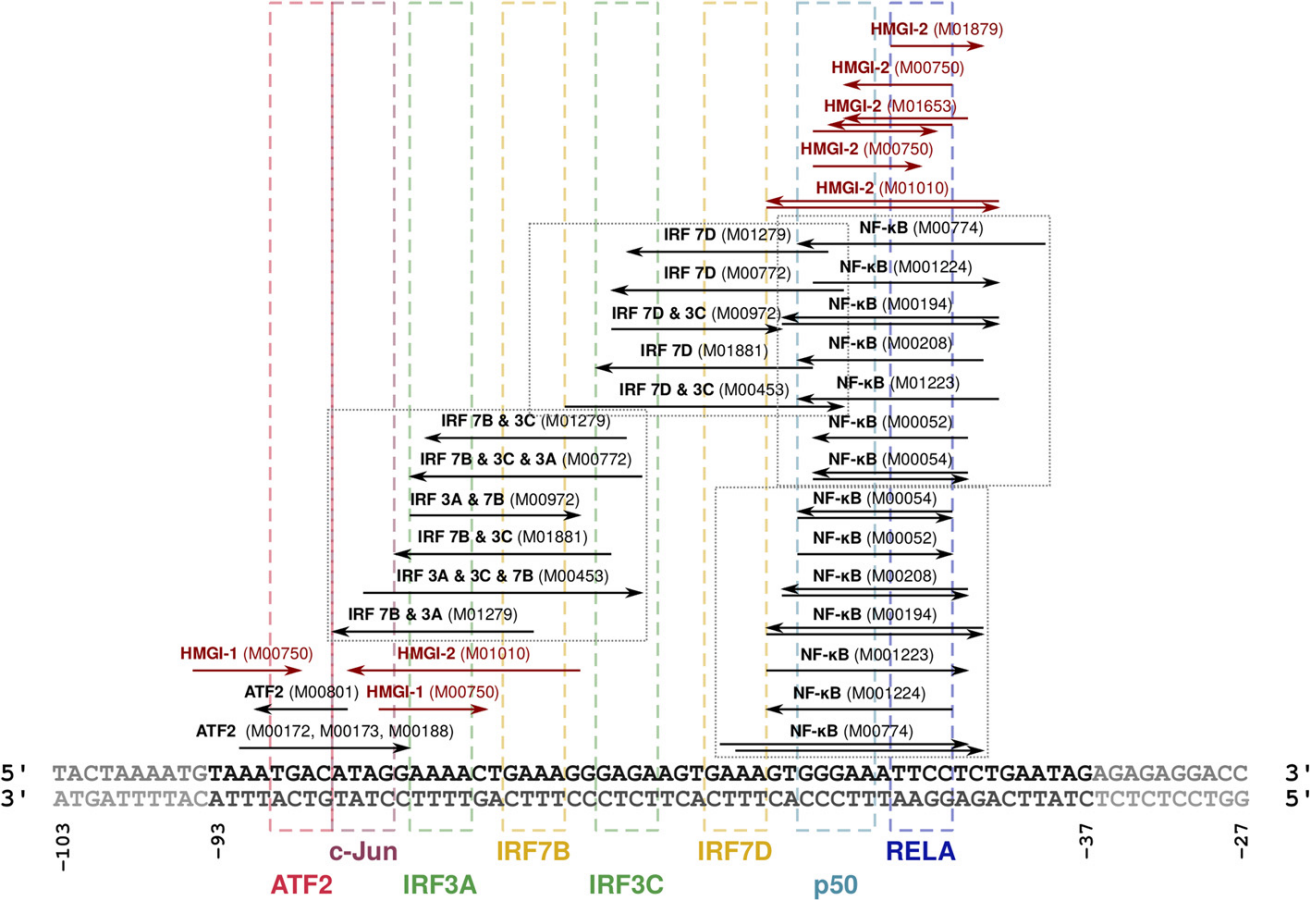
Motif hits along the enhanceosome sequence. For the selected 20 TRANSFAC PWMs, their hits (including orientation) are presented along the enhanceosome sequence. Colored rectangles represent binding sites of the enhanceosome activators as reported by [4]. It should be noted that in most cases a PWM represents binding affinity for a dimer

For our experiment we selected 20 TRANSFAC PWMs that represent proteins which are involved in enhanceosome assembly (Additional file 1: Table S1). Figure 1 presents the enhanceosome sequence together with positions and orientation of the PWM hits along that sequence. Among all predictions returned by TACO, some did not match the structure of the dimer binding the enhanceosome as known from the literature. Since the 3D structure of the enhanceosome may have slightly affected the offset of the involved dimers through allosteric effects, in selecting the predictions we allowed a ±2 bp margin on the predicted offset with respect to the offset derived from the published enhanceosome structure. In this way we ended up with 137 predictions for the human enhanceosome (Additional file 2). A similar table with mouse predictions was also obtained (Additional file 2).

In human the total number of different predicted dimer structures was 16, while for mouse it was 7. They covered 36 for human, and 14 for mouse cell types. One human cell type (Treg_Wb78495824) had 12 different dimers predicted (75 % of all dimers). In mouse the cell type with the largest number of predicted dimers (5) was Treg (71 % of all dimers).

**Fig. 2:**
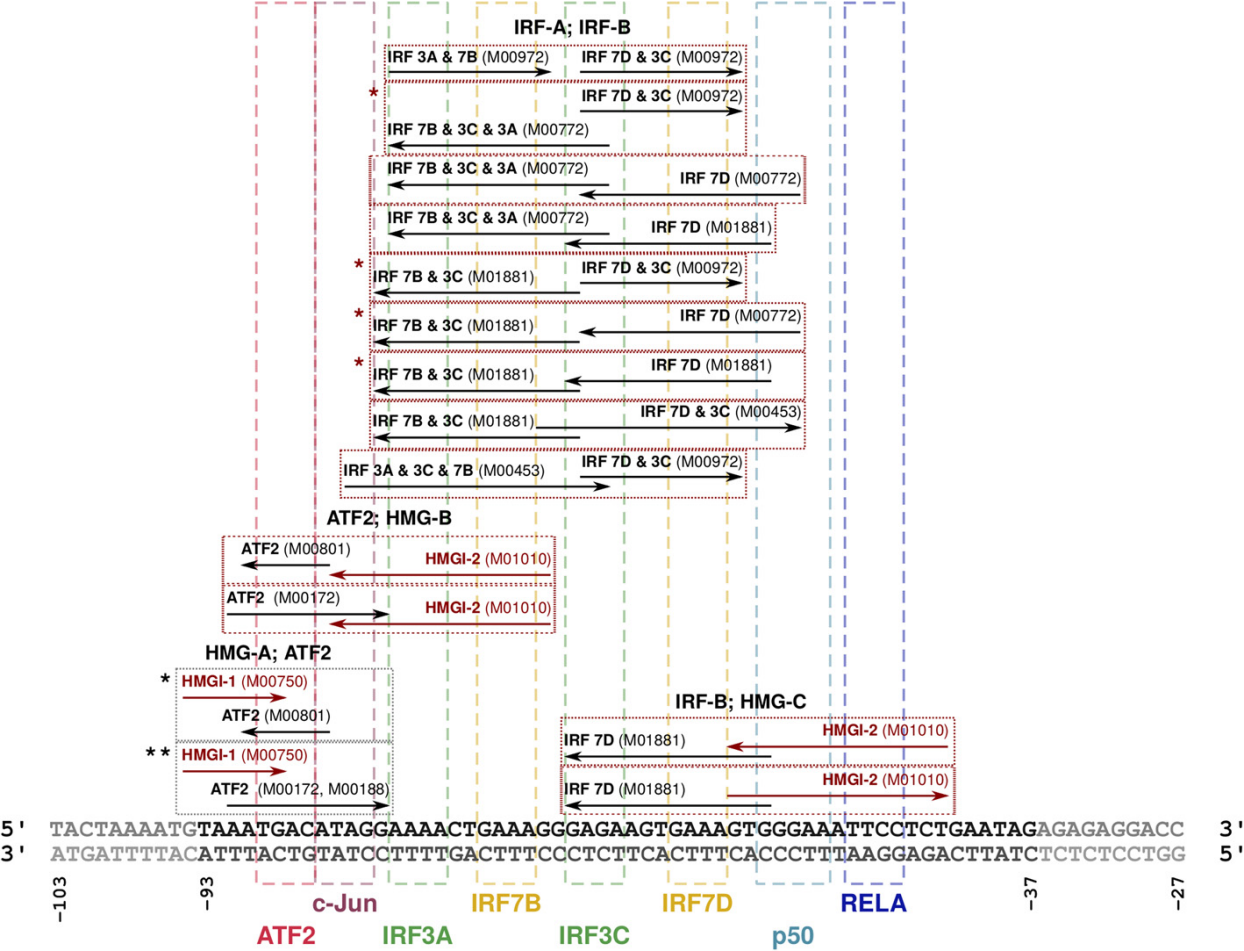
Predicted dimers mapped to the enhanceosome sequence. Positions of predicted dimers are mapped to the human enhanceosome sequence. Each rectangle represents one dimer (except the one with two asterisks next to it, which actually represents two dimers of exactly the same structure, due to two motif occurrences that represent ATF2). Red rectangles indicate the cases where motifs found in the enhanceosome have 1 bp wider spacing than the genome-wide dimers; gray rectangles indicate the cases where the spacing matches. Dimers are grouped according to their type into four groups. Dimers that were predicted both in human and in mouse are marked with an asterisk. Position of each motif composing a dimer is indicated with an arrow; orientation of the arrow corresponds to the orientation of the occurrence of this motif

Following the positions of hits in the enhanceosome we grouped our predictions into four groups: HMG-A; ATF2 (representing the enhanceosome dimer consisting of HMG I, the leftmost binding, with ATF-2/c-Jun activator), ATF2; HMG-B (representing the dimer consisting of ATF-2/c-Jun activator with HMG I, the middle binding, IRF-A; IRF-B (homodimer of IRF proteins), and IRF-B; HMG-C (representing the dimer consisting of IRF and HMG I, the rightmost binding). Figure 2 shows positions of predicted dimers mapped to the enhanceosome sequence; it also shows the above mentioned four groups of predicted dimers. Note that 13 of these 16 genome-wide overrepresented dimer structures were characterized by a motif spacing 1 bp tighter than in the original enhanceosome.

Table 1 shows the breakdown of predicted dimers in the human enhanceosome with respect to dimer types. It also indicates the number of cells a given dimer was predicted in. In boldface are shown dimers that were also predicted in mouse. A similar table for mouse predicted dimers is given in Additional file 1: Table S2. It is noticeable that only dimers found in the highest number of cell types were those predicted both in human and mouse enhanceosome. Dimer M00750; M00801 (of type HMG-A; ATF2) was predicted in 24 cell types in human (67 % of all human cell types that contained a predicted dimer) and in all the 14 cell types that contained a predicted dimer in mouse.

**Table 1:** Breakdown of predicted dimers in human cell types with respect to dimer types

HMG-A; ATF2	ATF2; HMG-B	IRF-A; IRF-B	IRF-B; HMG-C
**M00750; M00172** (22)	M01010; M00801 (6)	**M00972; M00772** (10)	M01010; M01881 (1)
**M00750; M00188** (22)	M01010; M00172 (3)	**M00972; M01881** (17)	M01881; M01010 (1)
**M00750; M00801** (24)		**M00772; M01881** (10)	
		**M01881; M01881** (10)	
		M00453; M01881 (1)	
		M00972; M00972 (6)	
		M00772; M00772 (2)	
		M01881; M00772 (1)	
		M00972; M00453 (1)	

In boldface are indicated the dimers that were also predicted in mouse. Numbers in parentheses show the number of cell types in which the dimer was predicted

It is interesting to notice that in both cases when a given dimer type was predicted in the largest number of cell types (HMG-A; ATF2 and IRF-A; IRF-B) the cell types related to a given dimer are very much the same. Table 2 illustrates this situation – all rows, except the last two, are identical. A very similar property holds for the dimer type IRF-A; IRF-B (Additional file 1: Table S3).

**Table 2:** Dimers from the HMG-A; ATF2 group predicted in various human cell types

M00750; M00172	M00750; M00188	M00750; M00801
HMVEC-dAd	HMVEC-dAd	HMVEC-dAd
M-CD14+_RO01746	M-CD14+_RO01746	M-CD14+_RO01746
HMVEC-dBl-Neo	HMVEC-dBl-Neo	HMVEC-dBl-Neo
Th2	Th2	Th2
Th1	Th1	Th1
HUVEC	HUVEC	HUVEC
Th2_Wb33676984	Th2_Wb33676984	Th2_Wb33676984
Th1_Wb33676984	Th1_Wb33676984	Th1_Wb33676984
HMVEC-LBl	HMVEC-LBl	HMVEC-LBl
HMVEC-LLy	HMVEC-LLy	HMVEC-LLy
CD34+_Mobilized	CD34+_Mobilized	CD34+_Mobilized
Th2_Wb54553204	Th2_Wb54553204	Th2_Wb54553204
HMVEC-dNeo	HMVEC-dNeo	HMVEC-dNeo
NB4	NB4	NB4
Th17	Th17	Th17
HRGEC	HRGEC	HRGEC
HMVEC-dLy-Neo	HMVEC-dLy-Neo	HMVEC-dLy-Neo
HMVEC-dBl-Ad	HMVEC-dBl-Ad	HMVEC-dBl-Ad
Treg_Wb78495824	Treg_Wb78495824	Treg_Wb78495824
HPAEC	HPAEC	HPAEC
HMVEC-dLy-Ad	HMVEC-dLy-Ad	HMVEC-dLy-Ad
GM12864	GM12864	GM12864
GM12865		GM12865
		GM06990

Note that M-CD14+_RO01746 is an abbreviation for Monocytes-CD14+_RO01746

### Predicted dimer binding sites are enriched in regulatory states

In order to check whether positions of instances of predicted dimers are enriched in chromatin states associated with regulatory function, we used the data deposited from the ENCODE Project for Broad ChromHMM [14]. In this version there are ChromHMM data (.bed files) available for 11 human cell types, out of which four cell types were present in our study: GM12878, HMEC, HUVEC and K562. In total, 11 dimers were predicted in the above mentioned cell types (Table 3).

**Table 3:** Human dimer instances located within a gene

Dimer-type	Dimer	Within-gene	Total	Percent
	M00750; M00172	1105	2029	54 %
HMG-A; ATF2	M00750; M00188	1141	2123	53 %
	M00750; M00801	2171	3956	54 %
ATF2; HMG-B	M01010; M00801	2344	4499	52 %
	M01010; M00172	1479	2783	53 %
IRF-A; IRF-B	M00972; M00772	395	707	55 %
	M00972; M01881	855	1471	58 %
	M00772; M01881	357	633	56 %
	M01881; M01881	695	1238	56 %
	M00453; M01881	46	73	63 %
	M00972; M00972	358	624	57 %
	M00772; M00772	95	187	50 %
	M01881; M00772	122	211	57 %
	M00972; M00453	37	63	58 %
IRF-B; HMG-C	M01010; M01881	738	1273	57 %
	M01881; M01010	239	457	52 %

Predictions are referred to by their hypothesis_id number. For a given cell type the data from ChromHMM for that cell type assigns, to each position in open chromatin, one of the 15 possible HMM states that describe predicted state of activity for that position. A dimer instance may have more than one state assigned to the genomic locus it covers. In order to assign a unique state to each instance, we decided to choose the state of the midpoint of the instance as representative.

**Fig. 3:**
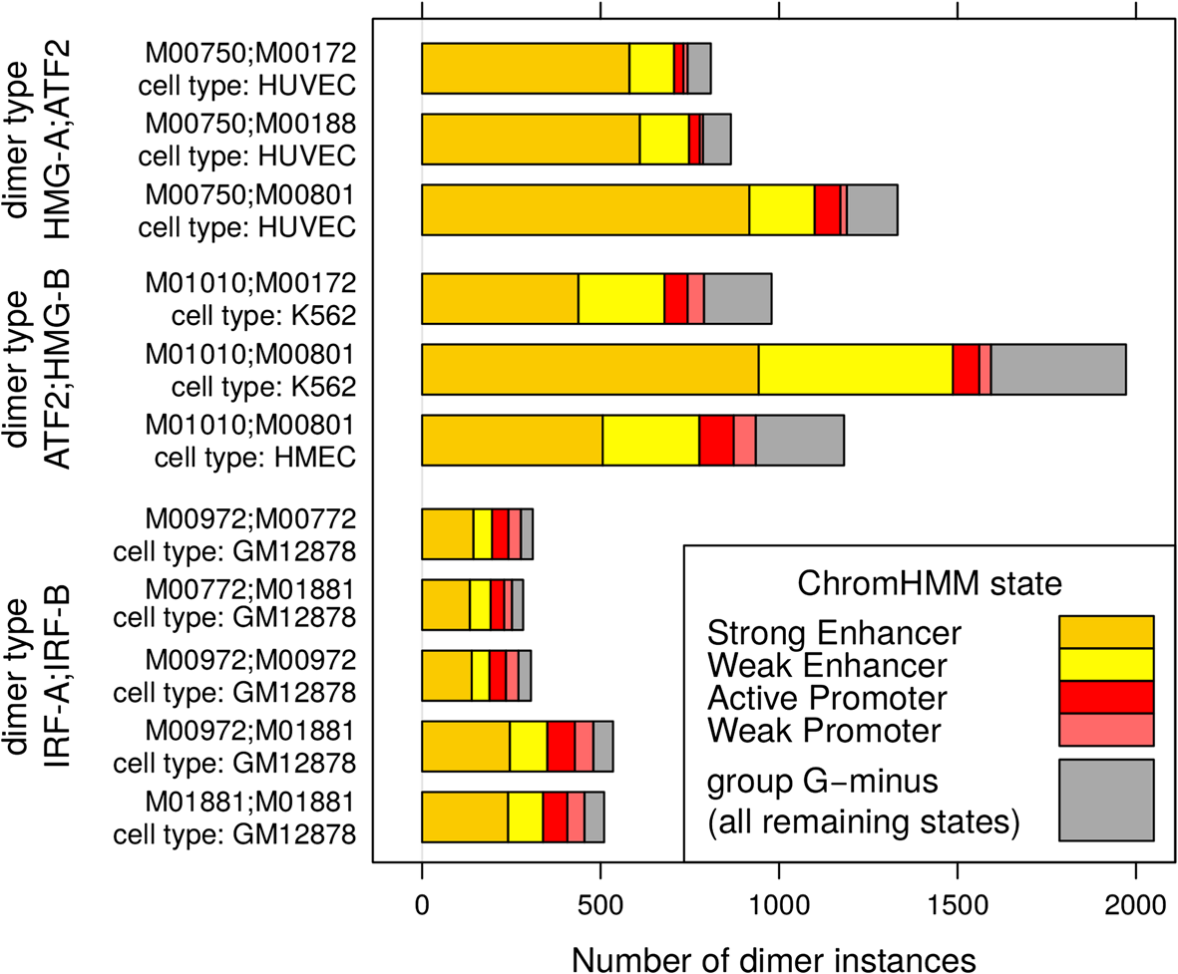
Breakdown of ChromHMM states with respect to dimer occurrences. For each predicted dimer in four human cell types: HUVEC, K562, HMEC, and GM12878, a breakdown is shown between ChromHMM regulatory states (G-plus), split into four groups, and the remaining states that compose the G-minus group. X-axis shows the number of dimer occurrences

In order to help assessing whether dimer instances are enriched in regulatory states related to promoter/enhancer elements we divided the set of all states into two groups: G-plus group consisting of: ‘Strong_Enhancer’, ‘Active_Promoter’, ‘Weak_Promoter’, and ‘Weak_Enhancer’, with G-minus consisting of the remaining states. Figure 3 shows a histogram of ChromHMM states assigned to dimer occurrences, where group G-minus forms one category, and G-plus is split into four subgroups, as indicated above. It is quite clear that overwhelming majority of dimer instances is assigned a regulatory state in G-plus. In order to assess a *p*-value of obtaining at least that many occurrences in G-plus group, this could be considered as a binomial process. A more detailed statistics is given in (Additional file 1: Table S4).

### Dimer instances are mainly located within introns

**Fig. 4:**
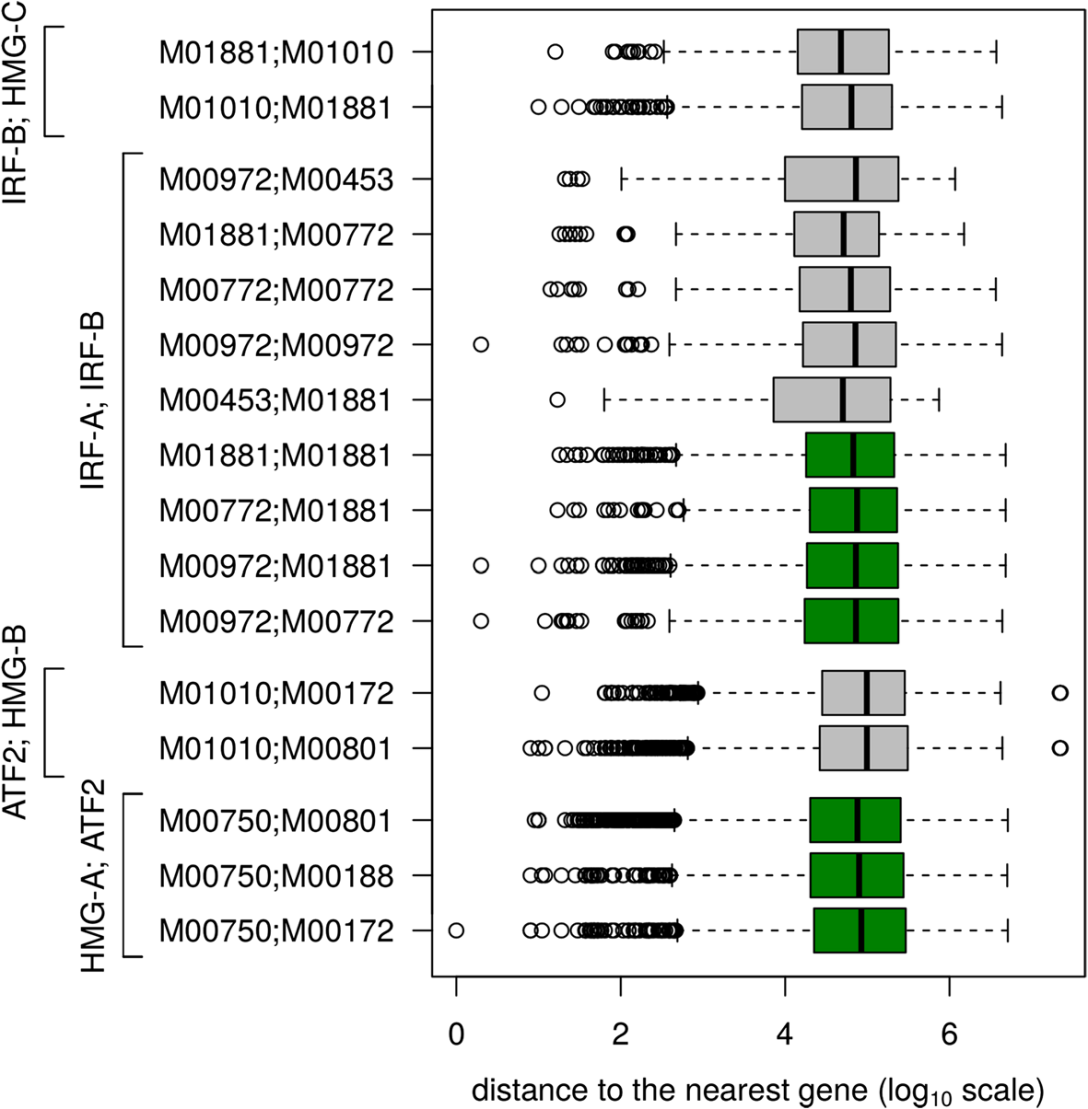
Distribution of distances from a dimer instance to the closest gene. For each predicted dimer in human cell types, the distribution of basepair distances from a dimer instance to the closest gene is shown as a boxplot. Grouping of dimers into dimer types is indicated. The boxplots for dimers that were predicted both in human and in mouse are shown in green

Next we investigated how far are located predicted dimer instances with respect to a nearest gene. Table 3 shows, for each predicted dimer, the total number of instances (in all cell types where the dimer was predicted) and the number of instances that are located within a gene. It clearly follows from that table that for all dimers most of their instances are located within a gene, which must be an intron, since we have masked all the exons. Distribution of the distances from a dimer instance to the closest gene is shown in Fig. 4. Observe that dimer instances that are not located within a gene are rather far from the nearest gene (median ranging from 46 to 95 kbp, depending on the dimer). Same observation holds for mouse as well, but here the spectrum of medians is bit smaller, ranging from 62 to 80 kbp.

### Dimer instances located near Transcription Start Site of a gene are enriched for being part of a computationally predicted enhancer

As an additional sanity check, we selected all 2663 human genes that contain within 10 kb for their Transcription Start Site (TSS) at least one instance of a predicted dimer. We restricted our attention to only those genes that contain at least three instances within the 10 kb distance from their TSS. In this way we reduced the number of genes to 70. Next we applied a computational tool, Billboard [15], to predict enhancers within the 20 kb area around TSS of each of the 70 genes. The salient feature of Billboard is that, with the help of an informant species, it discovers evolutionarily conserved arrangements of TFBS. Within a short window (here 50 bp) arbitrary permutations of TFBSs are allowed. For this experiment we chose five informant species: mouse, rat, macaque, opossum, and chicken. Of the 70 human genes, six did not have any ortholog in any of the five informant species, so they had to be removed from further analysis. The remaining 64 genes had in total 205 dimer instances within ±10 kb of the TSS. We run Billboard on these 64 human genes to find positions of predicted enhancers. As a result 183 dimer instances (89 % of all considered instances) ended up within a predicted enhancer (*p*-value 2.6*e*−05). On average, the number of informant species supporting a given Billboard prediction was 2.24. Additional file 3 contains information about the selected human genes and the outcome of this computational experiment.

### Gene Ontology enrichment of dimer selected genes

In order to elucidate a potential regulatory function of predicted dimers we investigated genes which are located in the neighbourhood of dimer instances. A gene is said to be *selected* by a dimer instance if it is the closest gene to that instance: either the instance is located within the gene, or else the distance between the midpoint of the instance and the closest nucleotide position within the gene is minimal (upstream or downstream). We group the selected genes, for a given dimer, into 7 sets: those that contain an instance of the dimer within its introns (denoted Q0), and those located within 100, 500, 1,000, 5,000, 10,000, and 50,000 bp from an instance of the dimer are denoted Q1, Q2, Q3, Q4, Q5, and Q6, respectively.

In addition to this, we also performed for each dimer a global analysis consisting of taking as Qx (*x*=0,1,…,6) set union of all sets Qx over all considered cell types.

Below we discuss enriched GO terms for dimer selected genes, separately for each of the four dimer types. We mainly focus on Q0 sets of genes since for these genes there were the most statistically significant GO enrichment terms. Here we consider all GO enriched terms for all gene categories having enrichment with FDR rate <0.01 (Additional files 4 and 5). Under this threshold there were no enriched terms for the dimers of type IRF-B; HMG-C.

**Fig. 5:**
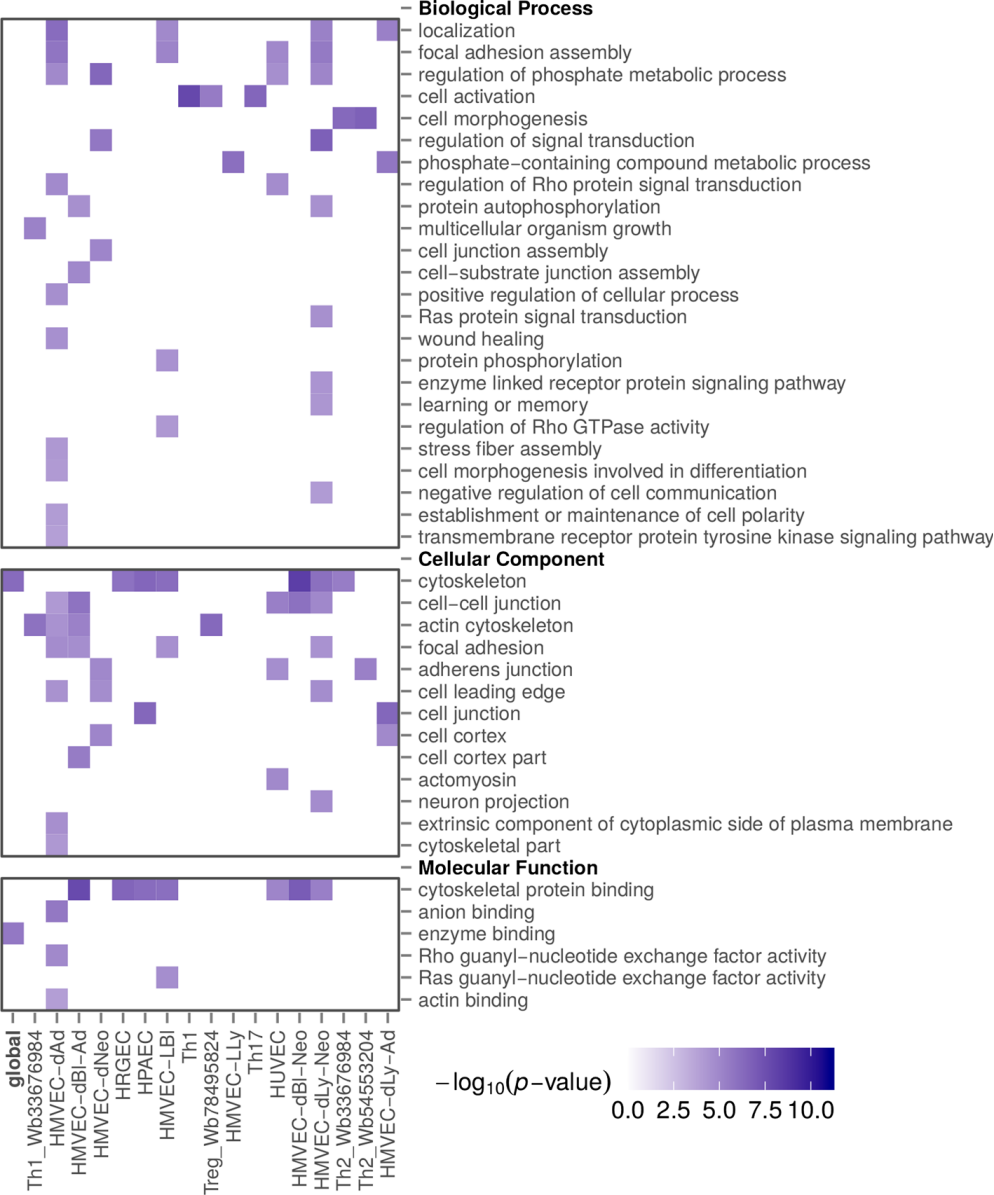
Heat map for Gene Ontology enriched terms. Enriched Gene Ontology terms associated with genes that contain instances of the dimer M00750; M00172, shown as a heatmap. This dimer is of type HMG-A; ATF2. We show enrichment *p*-values for the global instances (set union of all instances of the same dimer in all cell types) and for each of the 17 human cell types in which the dimer was predicted

For dimers of type HMG-A; ATF2 there were only 5 cell types with many enriched terms for genes in set Q0 (HMVEC-dAd, HMVEC-dBl-Ad, HMVEC-dLy-Neo, HMVEC-dNeo, HMVEC-LBl), all of them being blood microvascular endothelial cells. The biological process (BP) enriched terms for Q0 genes selected by dimers of this type are: ‘regulation of Rho protein signal transduction’, ‘focal adhesion assembly’, ‘regulation of phosphate metabolic process’, ‘establishment or maintenance of cell polarity’, and ‘actin cytoskeleton organization’. For cellular component (CC) domain the enriched terms include: ‘focal adhension’, ‘cytoskeleton’, ‘cell cortex’, ‘neuron projection’, and ‘cell-cell junction’. Finally, for molecular function (MF) domain we have the following enriched terms: ‘cytoskeletal protein binding’, ‘Ras/Rho guanyl-nucleotide exchange factor activity’, and ‘protein tyrosine kinase activity’. It is also noticeable that the set of genes located within 500 bp of a dimer instance (Q2 query) has enriched a BP term ‘positive regulation of tyrosine phosphorylation of STAT protein’. As an illustration, Fig. 5 shows a complete set of enriched GO terms for just one dimer (M00750; M00172) of type HMG-A; ATF2 for all the 17 human cell types that this dimer was predicted.

An interesting collection of enriched terms is for genes selected by dimers of type ATF2; HMG-B. For BP domain the enriched terms include: ‘axon guidance’, ‘cell junction assembly’, ‘regulation of signal transduction’, ‘transmembrane receptor protein tyrosine kinase signalling pathway’, ‘wound healing’, and ‘actin filament organization’. Enriched GO terms for CC domain include: ‘neuron projection’, ‘adherence junction’, and ‘actin cytoskeleton’, while terms for MF domain are: ‘actin binding’, and ‘Rho/Ras guanyl-nucleotide exchange factor activity’.

The third dimer type, IRF-A; IRF-B, does not have a very pronounced set of enriched terms for Q0 genes. Instead it has an interesting set of enriched terms for Q2–Q3 sets of genes. Notably, the BP enriched term ‘ISG15-protein conjugation’ is enriched for all queries Q1–Q5. A BP term ‘negative regulation of type I interferon production’ is enriched for many cell types. Likewise the BP terms ‘defense response to virus’, ‘defense response to other organism’, and ‘NLS-bearing protein import into nucleus’ are enriched for Q2 and Q3 queries. Also query Q6 has a very wide spectrum of BP enriched terms. These are mainly involved in defense response and in the adaptive immune system.

### Enriched GO terms for Q0 and Q6 selected genes are similar between human and mouse

In order to assess similarity of enriched terms between human and mouse we performed the following experiment. For a given dimer *D* that was predicted in human and mouse cell types we consider all pairs of cell types (*C*_*H*_,*C*_*M*_) such that *D* was predicted in human cell type *C*_*H*_, as well as in mouse cell type *C*_*M*_. For *i*=0,…,6, let $G_{H}^{(i)}$ denote the Q*i* selected genes that are also selected by dimer *D* in *C*_*H*_. In a similar way we define genes $G_{M}^{(i)}$ selected in *C*_*M*_. Let $T\left (G_{H}^{(i)}\right)$ and $T\left (G_{M}^{(i)}\right)$ denote the set of all GO terms enriched for genes in $ G_{H}^{(i)}$ and in $G_{M}^{(i)}$, respectively. We assess statistical significance of the intersection of these two sets by performing Fisher’s exact test, considering as universe the set of all GO terms that can be assigned to human or mouse genes, correspondingly.

We address here only dimers of type HMG-A; ATF2 and IRF-A; IRF-B. Only GO terms for genes selected by Q0 and Q6 queries had statistically significant intersections between human and mouse. Table 4 contains mean values for these distributions. We also present there *p*-values for Fisher’s exact test for global instances for each dimer and each pair of cell types (Additional file 6). It clearly follows from that table that enriched GO terms for Q0 selected human and mouse genes are quite similar to each other for dimers of type HMG-A; ATF2. It is less so for dimers of type IRF-A; IRF-B and for Q6 selected genes, but still in some cases the enriched terms are reasonably similar.

**Table 4:** *p*-values (minus log to the base 10 of reported *p*-values) for the Fisher’s exact test for similarity of GO terms enriched for human and mouse selected genes. Here we report only Q0 and Q6 selected genes

Dimer type	Dimer	*p*-value Q0 global	Best Q0 *p*-value	*p*-value Q6 global	Best Q6 *p*-value
HMG-A; ATF2	M00750; M00172	78.96	80.15 (HdL/416)	0.35	3.31 (HdA/Spl)
HMG-A; ATF2	M00750; M00188	72.44	40.97 (HLB/416)	1.51	1.73 (HLL/416)
HMG-A; ATF2	M00750; M00801	62.74	159.94 (HdA/CH)	0.37	14.16 (Th2/Thy)
IRF-A; IRF-B	M00772; M01881	0.61	2.01 (GM/Spl)	3.02	3.01 (CD/A20)
IRF-A; IRF-B	M00972; M00772	0.59	2.59 (GM/TRA)	13.22	10.42 (CD/TR)
IRF-A; IRF-B	M00972; M01881	7.27	9.22 (Mon/Bcl)	2.12	16.86 (ThW/TR)
IRF-A; IRF-B	M01881; M01881	7.94	10.16 (ThW/Bcl)	8.55	5.41 (CD/TR)

Column ‘*p*-value Q0 global’ refers to *p*-value of Fisher’s exact test for globaly selected genes (set union of all genes selected by the same dimer in different cell types). Column ‘best Q0 *p*-value’ shows minus log to the base 10 of the best reported *p*-value, names in brackets are cell types in human/mouse for which this *p*-value was achieved. Same explanation applies to Q6. Here are the abbreviations of the cell type names: (human) HdL = HMVEC-dLy-Neo, HdA = HMVEC-dAd, HLB = HMVEC-LBl, HLL = HMVEC-LLy, GM = GM12865, CD = CD2+_RO01778, Mon = Monocytes-CD14+_RO01746, ThW = Th2_Wb54553204; (mouse) 416 = 416B, Spl = Spleen, CH = CH12, Thy = Thymus, TRA = TReg-Activated, TR = TReg, Bcl = B-cell_(CD19+)

### Q0 and Q6 selected genes for human and mouse are in close homology

In the same spirit we assessed similarity of genes selected by the same dimer in human and in mouse. Here we have to preprocess the data so that Fisher’s exact test is applicable. Given a dimer *D* and selected genes: *G*_*H*_, in human and *G*_*M*_, in mouse, the main issue is how to interpret intersection of *G*_*H*_ and *G*_*M*_. For this end, we use orthology relation between human and mouse genes. Since this relation is of many-to-many kind, it follows that we should take the smallest partition of *G*_*H*_ and the smallest partition of *G*_*M*_, so that if two genes *g*_1_,*g*_2_ in one set are orthologs to a common gene in another set, then *g*_1_,*g*_2_ belong to the same block of the partition. See Methods section for a more detailed description. Now, intersection between *G*_*H*_ and *G*_*M*_ is interpreted as the number of partition blocks, say of *G*_*H*_, that have a corresponding partition block in *G*_*M*_ related by at least one orthology relation between members of these blocks. The universe is then taken as the number of equivalence classes in the set union of human and mouse genes.

Table 5 contains information about *p*-values assigned to Fisher’s exact test for similarity of Q0 and Q6 selected human/mouse genes under global dimer approach, as well as information about a pair of cell types (human/mouse) that have the best *p*-value for the given dimer. It clearly follows from this table that Q0 selected genes for dimers of type HMG-A; ATF2 exhibit higher similarity between human and mouse than for dimers of type IRF-A; IRF-B. However, for the latter dimers similarity between selected genes is still quite clearly visible. Additional file 7 contains detailed information about *p*-values assigned to Fisher’s exact test for each dimer and each pair of human-mouse cell types.

**Table 5:** *p*-values (minus log to the base 10 of reported *p*-values) for the Fisher’s exact test for similarity of dimer selected genes between human and mouse. Explanation and abbreviations are as in Table 4. Addtional abbreviations: (human) HU = HUVEC, Th1 = Th1_Wb33676984; (mouse) Zh = ZhBTc4

Dimer type	Dimer	*p*-value Q0 global	Best Q0 *p*-value	*p*-value Q6 global	best Q6 *p*-value
HMG-A; ATF2	M00750; M00172	32.93	18.1 (HLB/416)	2.46	2.06 (HU/416)
HMG-A; ATF2	M00750; M00188	35.02	19.54 (HdL/416)	2.17	2.87 (HU/416)
HMG-A; ATF2	M00750; M00801	299.07	60.35 (HU/Zh)	121.63	24.76 (Th1/Spl)
IRF-A; IRF-B	M00772; M01881	11.10	6.79 (CD/A20)	2.56	3.05 (ThW/A20)
IRF-A; IRF-B	M00972; M00772	10.91	8.32 (Th1/TRA)	0.75	1.26 (Th1/TRA)
IRF-A; IRF-B	M00972; M01881	65.49	19.49 (Th2/TR)	12.46	8.28 (Th2/A20)
IRF-A; IRF-B	M01881; M01881	50.10	17.43 (CD/A20)	11.9	7.09 (Th1/Bcl)

## Discussion

Of the 16 predicted dimers in the human enhanceosome, 7 dimers were constructed from an architectural protein of High Mobility Group I(Y) (HMG I) and one of the two transcriptional activators: ATF-2/c-Jun and interferon regulatory factor (IRF). In total these heterodimers fall into three types, depending on their binding location in the enhanceosome: the leftmost enhanceosome binding position of HMG I (HMG-A) with ATF-2/c-Jun, ATF-2/c-Jun with the middle enhanceosome binding position of HMG I (HMG-B), and IRF with the rightmost enhanceosome binding position of HMG I (HMG-C). The remaining 9 dimers constituted homodimeric arrangement of IRF with itself. It is remarkable that nuclear factor kappa-light-chain-enhancer complex of activated B cells (NF- *κ*B) did not show up in this analysis. It should be kept in mind that dimers predicted by TACO are only those that show overepresentation in terms of binding instances in a particular cell type over the background. Hence, if a certain dimer was not predicted it does not mean that this dimer configuration is not plausible.

In total the dimers were predicted in 36 human cell types: two from ENCODE Tier 1 (GM12878 and K562), three from ENCODE Tier 2 (CD20+_RO01778, HUVEC, and Monocytes-CD14+_RO01746), and the rest of cell types are from ENCODE Tier 3. Most of the cells with predicted dimers are from blood tissues: monocytes, B cells, B-lymphocyte, T cells, T regulatory cells, and T helper cells – all of them of normal karyotype, but also some diseased cells, mainly leukemia (NB4, K562, and CMK). It is noticeable that for all types of dimers, except ATF2; HMG-B, the predicted cell types that contain the dimer are mainly blood cells. For both dimers of type ATF2; HMG-B the predicted cell types are leukemia (K562), prostate (normal cell, PrEC), and epithelium (SAEC, RPTEC, HMEC, and HEEpiC).

We also performed a similar analysis in mouse cell types. We have found 7 dimers present in 14 cell types. It is remarkable that the structure (motif pair, offset and mutual orientation) of all these dimers matched exactly the structure of dimers predicted in human. It is also noticeable that the 7 dimers predicted both in human and in mouse exhibit the highest number of human cells containing them.

Interestingly, 13 dimer structures predicted in human out of 16, were characterized by a motif spacing 1 bp tighter than in the original enhanceosome. What is more interesting is that the remaining 3 dimers (the ones that have the same offset as in the original enhanceosome) are all of the same type HMG-A; ATF2 and are therefore located on the enhanceosome leftmost side of the sequence. All of the above mentioned 13 dimer structures are located inside the enhanceosome. An additional support that we are not discussing here is that the above mentioned 3 dimers are predicted in mouse with exactly the same offset, and the other 4 mouse dimers, having the same offset as in human, turn out to be 1 bp tighter than in the original mouse enhanceosome. A possible mechanistic explanation of this phenomenon is that a 1 bp wider offset between TFs that are located inside of the big protein complex which binds the enhanceosome is caused by the overall three-dimensional structure of the complex, with weaker constraints imposed on the sides of the enhanceosome complex.

As a sanity check we investigated ChromHMM states assigned to genomic dimer instances. In an overwhelming number of cases these instances were assigned to an enhancer or promoter state. More than half of the instances are located within a gene (in introns). Moreover, using an enhancer annotation tool, Billboard, we searched the ±10 kb area around TSS of human genes that contain at least three dimer instances. With help of five informant species, we concluded that in 89 % of the 205 instances they were included in a predicted enhancer. This supports the hypothesis that the predicted dimers, being part of the human enhanceosome, are also utilized in the genome by other enhancers.

In order to analyze genes which contain in their vicinity a dimer instance, we defined 7 groups, which we call queries: genes containing a dimer instance (Q0), and queries Q1 through Q6 that consist of genes that the closest distance to a dimer instance is less than 100, 500, 1000, 5 000, 10,000, and 50,000 base pairs. Next, for each dimer we performed Gene Ontology analysis for each of the above mentioned queries. We have also compared enriched GO terms for similarity between human and mouse. The performed Fisher’s exact test suggests that the enriched terms for queries Q0 and Q6 are significantly similar between human and mouse. Even stronger similarity is observed for comparison of human/mouse genes selected by these two queries. For this we adopted Fisher’s exact test by utilizing the orthology relation between human and mouse genes.

## Conclusions

We have performed a comprehensive search of overrepresented dimers composed of transcription factors that constitute the human enhanceosome. We searched over many cell types, both in human and in mouse. The predicted dimers were grouped into 4 types, according to their binding position in the enhanceosome: HMG-A; ATF2, ATF2; HMG-B, IRF-A; IRF-B, and IRF-B; HMG-C. Here HMG-{A, B, C} represents three binding sites of the architectural protein HMG I in the enhanceosome. No other combinations of TFs and/or architectural proteins were predicted. The majority of cell types in which these dimers were discovered were various kinds of blood tissues.

Despite the fact that we allowed for dimer spacing variability up to ±2 bp comparing to the enhancesome sequence, most of the predicted dimers were exactly 1 bp wider than suggested by the motifs within the enhanceosome. The remaining 3 dimer predictions were of the same type HMG-A; ATF2, located on the side of the enhanceosome. Since in most of the cases the predicted dimer binding sites were associated with a state of regulatory character, we assumed that the role of these dimers is being a part of enhancers other than the enhanceosome. We observe that dimer instances are preferentially located within introns of genes and that those instances that are located near TSS of a gene are preferentially included in computationally predicted enhancers (*p*-value <2.6*e*−05).

Gene Ontology analysis for the groups of genes that are located near dimer binding sites shows that these dimers are possibly involved in various kinds of activities: the first two types seem to be involved in regulation of signal transduction and in Ras guanyl-nucleotide exchange factor activity. On the other hand, the third type of dimers is mainly involved in defense response and in the adaptive immune system. Predicted dimers for mouse, even though they form a subset of those in human, have similar properties. The enriched Gene Ontology terms for the corresponding human and mouse genes are similar. Also the corresponding genes, related by orthology relation, are similar between human and mouse.

## Methods

### Human and mouse enhanceosome

We took as the human enhanceosome the 57 bp piece of DNA located on chromosome 9 (positions 21077989 to 21078045 in genome assembly hg19), which is 44 bp upstream of the human interferon beta 1 gene *IFNB1* (Ensembl ID: ENSG00000171855). Search of a homologous piece in the mouse genome reveals a region of very high similarity on chromosome 4 (*E*-value =3.7*e*−33) that is located 101 bp upstream of mouse interferon beta 1 gene *Ifnb1* (Ensembl ID: ENSMUSG00000048806). We took for the analysis a sequence from the mouse genome (assembly mm9) of the same length (57 bp). Human and mouse enhanceosomes are almost identical: except for the first position and the last four positions, they differ by only two mismatches.

Here are the corresponding enhanceosome sequences: **Human:**TAAATGACATAGGAAAACTGAAAGGGAGAAGTGAAAGTGGGAAATTCCTCTGAATAG**Mouse:**AAAATGACAGAGGAAAACTGAAAGGGAGAACTGAAAGTGGGAAATTCCTCTGAGGCA

### Selection of motifs

It should be noted that in TRANSFAC motif database, some TF motifs of binding sites already represent dimeric complexes. In the case of the enhanceosome, this happens for ATF-2+c-Jun dimer, as well as NF- *κ*B dimer (p50+RelA). We took these motifs without any further slicing. Also motifs for the two related TFs: IRF3 and IRF7 were difficult to discriminate, so we took all such motifs as representing any of the two TFs. In addition to this, we also considered High Mobility Group (HMG) proteins that function in the enhanceosome as architectural proteins which remove the intrinsic bend of the double helix and therefore allow binding of transcriptional activators [8].

A set of 27 motifs was initially selected for further analysis. We scan the human enhanceosome sequence with each motif with the motif score threshold set in such a way as to give the balanced threshold of 100, i.e. the threshold that makes the ratio of false positive rate to false negative rate at the level of 100 [16]. It turned out that of the 27 motifs only 20 had hits in the enhanceosome above the set threshold: there were 4 (out of 8) motifs representing the complex ATF-2+c-Jun; 5 (out of 5) motifs representing IRF3/IRF7; 7 (out of 8) representing NF- *κ*B complex; and 4 (out of 6) representing HMG motifs. TRANSFAC identifiers of the selected 20 motifs are reported in Additional file 1: Table S1.

### Generating TACO predictions

We collected DNase I hypersensitivity data for human (193 narrowPeak files, accounting for 105 cell types) and for mouse (123 narrowPeak files, accounting for 42 cell types). This data was generated at the University of Washington and obtained from the ENCODE Project, Genome Browser track wgEncodeUwDnase. The list of DNase I files for human and mouse and the list of corresponding cell types is given in Additional file 8.

Standalone tool TACO [11] was provided with all the datasets specified as WeaklySpecificDatasets. As in the default TACO setting, coding regions and repetitive elements were masked, individual DNase peaks were excluded if most of the underlying genomic sequence was masked (RegionMasking =Majority), up to 50,000 DNase peaks with top signalValue from each replicate were considered (RegionCount =50000). Deviating from the default settings, no lower bound was imposed for the number of instances of predicted dimer in the target dataset (TargetInstancesThreshold = 1).

### Finding nearest gene for a dimer binding site

Each dimer binding site was classified as either located within a gene (coding part of genes was masked, so it was located in an intron), or else a distance to the nearest gene (both upstream and downstream) was calculated. Here we take the midpoint of the dimer instance and calculate its distance to the nearest position of a gene (it would be a Transcription Start Site (TSS), if the gene is located downstream, or the end position of a gene, if that gene is located upstream). Then, for a given distance x (x is one of the distances 100, 500, 1,000, 5,000, 10,000, and 50,000) and for a given dimer binding site we report all genes such that the calculated distance from the dimer binding site is less than x.

### Gene Ontology enrichment analysis

Gene Ontology analysis for the selected genes was performed with help of Biopython [17] and Bio.Ontology module within it (Koziara K, Wilczynski B: Bio.Ontology – Python tools for enrichment analysis and visualization of ontologies, in preparation).

### Assessing similarity of dimer selected genes between human and mouse

In order to assess similarity of selected genes between human and mouse we have to preprocess the data. Let *G*_*H*_ and *G*_*M*_ be selected genes in human and mouse, respectively. Let *R*_*O*_ be a many-to-many gene orthology relation between human and mouse. We iteratively build partitions of *G*_*H*_ and *G*_*M*_ as follows. Initially all partition blocks in *G*_*H*_ and *G*_*M*_ are one-element and the orthology between blocks is given by *R*_*O*_. Iteratively, we join two partition blocks to form a new block (say X) in human (or in mouse) when they are linked by the orthology relation to a common block (say Y) in the other species. The new block X is linked by new orthology relation to Y. The process terminates when no new blocks to be jointed exist.

Fortunately, when we run this procedure on the data it terminates very quickly with only very few blocks having more than one element.

## Additional files

Additional file 1
**Supplementary tables.** Supplementary tables providing various kinds of additional information. Table S1 gives a list of selected PWMs. Table S2 shows predicted dimers in mouse. Table S3 contains dimers from IRF-A; IRF-B group predicted in various human cell types. Table S4 gives detailed statistics of ChromHMM states assigned to dimer instances. (PDF 43.3 kb)

Additional file 2
**Selected dimer predictions in human and mouse enhanceosome.** The table contains information on the correct dimer structure that fits the literature data on the enhanceosome. It also contains various details of the predictions, including structure of the dimer, name of the cell type, *p*-value of the prediction, and number of instances in open chromatin part of the genome. (XLS 87.5 kb)

Additional file 3
**Table of inclusion of dimer instances within Billboard predicted enhancers.** Presented are 64 human genes that contain within ±10 kb of their TSS at least three instances of predicted dimers. For each of these instances, we report the number of informant species in which the instance ended up within a predicted enhancer. (XLS 20 kb)

Additional file 4
**Table of Gene Ontology enriched terms.** Gene Ontology enriched terms with FDR <0.01 for predicted dimers of HMG-A; ATF2, ATF2; HMG-B and IRF-A; IRF-B types. Column ‘Cell_type’ contains information about enriched term in the indicated cell type. When this entry is empty it indicates that the enriched term is with respect to the union of of all dimer instances, taken over all cell types in which the dimer was predicted. (XLS 377 kb)

Additional file 5
**Visualization of Gene Ontology enriched terms.** Heatmap visualization of the Gene Ontology enriched terms with FDR <0.01 for predicted dimers. (PDF 60.1 kb)

Additional file 6
**Comparison of human and mouse Gene Ontology enriched terms.** Fisher’s exact test *p*-values for comparing human and mouse enriched Gene Ontology terms for the same dimer and for all pairs of cell types from human and mouse. (XLS 184 kb)

Additional file 7
**Comparison of human and mouse dimer selected genes.** Fisher’s exact test *p*-values for comparing human and mouse dimer selected genes for all pairs of cell types from human and mouse. (XLS 360 kb)

Additional file 8
**List of human and mouse DNase I hypersensitivity datasets considered.** (XLS 38 kb)

## Abbreviations

BP: biological process
CC: cellular component
GO: gene ontology
HMG: high mobility group
IRF: interferon regulatory factor
MF: molecular function
PRD: positive regulatory domain
PWM: position weight matrix
TF: transcription factor
TFBS: transcription factor binding site
TSS: transcription start site

## References

[1] Panne D (2008). The enhanceosome. Curr Opin Struct Biol.

[2] Ford E, Thanos D (2010). The transcriptional code of human ifn-beta gene expression. Biochim Biophys Acta.

[3] Escalante CR, Nistal-Villán E, Shen L, García-Sastre A, Aggarwal AK (2007). Structure of irf-3 bound to the prdiii-i regulatory element of the human interferon-beta enhancer. Mol Cell.

[4] Panne D, Maniatis T, Harrison SC (2007). An atomic model of the interferon-beta enhanceosome. Cell.

[5] Panne D, Maniatis T, Harrison SC (2004). Crystal structure of ATF-2/c-Jun and IRF-3 bound to the interferon-beta enhancer. EMBO J.

[6] Pan Y, Nussinov R. The Role of Response Elements Organization in Transcription Factor Selectivity: The IFN- *β* Enhanceosome Example. PLoS Comput Biol. 2011; 7(6). 10.1371/journal.pcbi.1002077 PMC3116919 21698143

[7] Arnosti DN, Kulkarni MM (2005). Transcriptional enhancers: Intelligent enhanceosomes or flexible billboards?. J Cell Biochem.

[8] Yie J, Merika M, Munshi N, Chen G, Thanos D (1999). The role of HMG I(Y) in the assembly and function of the IFN-beta enhanceosome. EMBO J.

[9] Balachandran S, Beg AA (2011). Defining emerging roles for nf- *κ*b in antivirus responses: revisiting the interferon- *β* enhanceosome paradigm. PLoS Pathog.

[10] Goh FG, Thomson SJP, Krausgruber T, Lanfrancotti A, Copley RR, Udalova IA (2010). Beyond the enhanceosome: cluster of novel *κ*b sites downstream of the human ifn- *β* gene is essential for lipopolysaccharide-induced gene activation. Blood.

[11] Jankowski A, Prabhakar S, Tiuryn J (2014). TACO: a general-purpose tool for predicting cell-type-specific transcription factor dimers. BMC Genomics.

[12] Jankowski A, Szczurek E, Jauch R, Tiuryn J, Prabhakar S (2013). Comprehensive prediction in 78 human cell lines reveals rigidity and compactness of transcription factor dimers. Genome Res.

[13] Wingender E (2008). The TRANSFAC project as an example of framework technology that supports the analysis of genomic regulation. Brief. Bioinformatics.

[14] Ernst J, Kellis M (2012). ChromHMM: automating chromatin-state discovery and characterization. Nat Methods.

[15] Wilczynski B, Dojer N, Patelak M, Tiuryn J (2009). Finding evolutionarily conserved cis-regulatory modules with a universal set of motifs. BMC Bioinformatics.

[16] Rahmann S, Müller T, Vingron M (2003). On the power of profiles for transcription factor binding site detection. Stat Appl Genet Mol Biol.

[17] Cock PJA, Antao T, Chang JT, Chapman BA, Cox CJ, Dalke A (2009). Biopython: freely available python tools for computational molecular biology and bioinformatics. Bioinformatics.

