# Supplementary material for: Enhanceosome transcription factors preferentially dimerize with high mobility group proteins
Source: BMC Syst Biol. 2016 Feb 4;10:14. doi: 10.1186/s12918-016-0258-3 (PMC4743414; doi:10.1186/s12918-016-0258-3)
Supplement: Additional file 1 — Supplementary tables. Supplementary tables providing various kinds of additional information. Table S1 gives a list of selected PWMs. Table S2 shows predicted dimers in mouse. Table S3 contains dimers from IRF-A; IRF-B group predicted in various human cell types. Table S4 gives detailed statistics of ChromHMM states assigned to dimer instances. (PDF 43.3 kb) [file 12918_2016_258_MOESM1_ESM.pdf]

Additional file 1: Supplementary tables

Aleksander Jankowski, Paulina Krawczyńska, Utsav Mathur, Jerzy Tiuryn

November 21, 2015

1 List of selected PWMs

ATF-2/c-Jun	IRF	NF-κB	HMG I(Y)
M00172	M00453	M00052	M00750
M00173	M00772	M00054	M01010
M00188	M00972)	M00194	M01653
M00801	M01279	M00208	M01879
	M01881	M00774	
		M01223	
		M01224	

Table 1: Selected TRANSFAC motifs for the transcriptional activators and architectural proteins.

2 Predicted dimers in mouse

HMG-A;ATF2	IRF-A;IRF-B
M00750;M00172 (2)	M00972;M00772 (2)
M00750;M00188 (1)	M00972;M01881 (8)
M00750;M00801 (14)	M00772;M01881 (3)
	M01881;M01881 (7)

Table 2: Break down of predicted dimers in mouse cell types with respect to dimer types. Numbers in parentheses show the number of cell types in which the dimer was predicted.

3 Predicted dimers from the IRF-A;IRF-B dimer type that are shared with mouse

M00972;M00772	M00972;M01881	M00772;M01881	M01881;M01881
Treg_Wb78495824<br>Th1<br>Th1_Wb33676984<br>Th1_Wb54553204<br>Th2<br>Th2_Wb54553204<br>CD20+_RO01778<br>GM12864<br>GM12865<br>GM12878	Treg_Wb78495824<br>Th1<br>Th1_Wb33676984<br>Th1_Wb54553204<br>Th2<br>Th2_Wb54553204<br>CD20+_RO01778<br>GM12864<br>GM12865<br>GM12878	Treg_Wb78495824<br>Th1<br>Th1_Wb33676984<br>Th1_Wb54553204<br>Th2<br>Th2_Wb54553204<br>CD20+_RO01778<br>GM12864<br>GM12865<br>GM12878	Treg_Wb78495824<br>Th1<br>Th1_Wb33676984<br>Th1_Wb54553204<br>Th2<br>Th2_Wb54553204<br>CD20+_RO01778<br>GM12864<br>GM12865<br>GM12878
Treg_Wb83319432	Treg_Wb83319432<br>Th17<br>CD4+_Naive_Wb11970640<br>Th2_Wb33676984<br>M-CD14+_RO01746<br>CD34+_Mobilized<br>GM06990	Th17	CD4+_Naive_Wb11970640

Table 3: Dimers from the IRF-A;IRF-B group predicted in various human cell types.These are all dimers in that group are predicted in both human and mouse. Note that M-CD14+_RO01746 is an abbreviation for Monocytes-CD14+_RO01746. Notice that in all rows above the double line show identical cell-types for each motif.

4 ChromHMM states assigned to dimer instances

Dimer-type	Cell	Dimer	SE	WE	AP	WP	GP	RC	PP	H	I	R	TT	TE	WT	GM	T
HMG-A;ATF2	HUVEC	M00750;M00172	581	125	26	12	**744**	2	3	25	16	5	3	0	11	**65**	**809**
HMG-A;ATF2	HUVEC	M00750;M00188	610	138	30	9	**787**	3	3	24	28	5	4	0	11	**78**	**865**
HMG-A;ATF2	HUVEC	M00750;M00801	917	183	72	18	**1190**	2	6	36	64	9	9	2	14	**142**	**1332**
ATF2;HMG-B	K562	M01010;M00172	438	241	65	46	**790**	13	6	28	60	14	51	8	9	**189**	**979**
ATF2;HMG-B	K562	M01010;M00801	943	544	74	33	**1594**	4	2	180	102	5	17	4	64	**378**	**1972**
ATF2;HMG-B	HMEC	M01010;M00801	506	271	96	62	**935**	15	10	33	92	15	58	9	15	**247**	**1182**
IRF-A;IRF-B	GM12878	M00972;M00772	144	52	46	35	**277**	0	2	12	6	1	2	3	7	**33**	**310**
IRF-A;IRF-B	GM12878	M00772;M01881	134	58	38	22	**252**	0	3	14	5	2	3	1	3	**31**	**283**
IRF-A;IRF-B	GM12878	M00972;M00972	139	50	46	35	**270**	0	2	14	8	1	3	3	4	**35**	**305**
IRF-A;IRF-B	GM12878	M00972;M01881	246	105	77	52	**480**	0	4	22	11	1	5	3	9	**55**	**535**
IRF-A;IRF-B	GM12878	M01881;M01881	241	98	68	48	**455**	1	3	18	13	2	6	1	11	**55**	**510**

Table 4: Detailed statistics of ChromHMM states assigned to dimer instances. *Abbreviations*: SE – strong enhancer; WE – weak enhancer; AP – active promoter; WP – weak promoter; RC – repetitive/CNV; PP – poised promoter; H – heterochromatin/low complexity region; I – insulator; R – repressed; TT – transcription transition; TE – transcription elongation; WT – weak transcription; GP – group G-plus; GM – group G-minus; T – total.
