# Supplementary material for: Enhanceosome transcription factors preferentially dimerize with high mobility group proteins
Source: BMC Syst Biol. 2016 Feb 4;10:14. doi: 10.1186/s12918-016-0258-3 (PMC4743414; doi:10.1186/s12918-016-0258-3)

human ATF2;HMG−B M01010;M00172, Q0

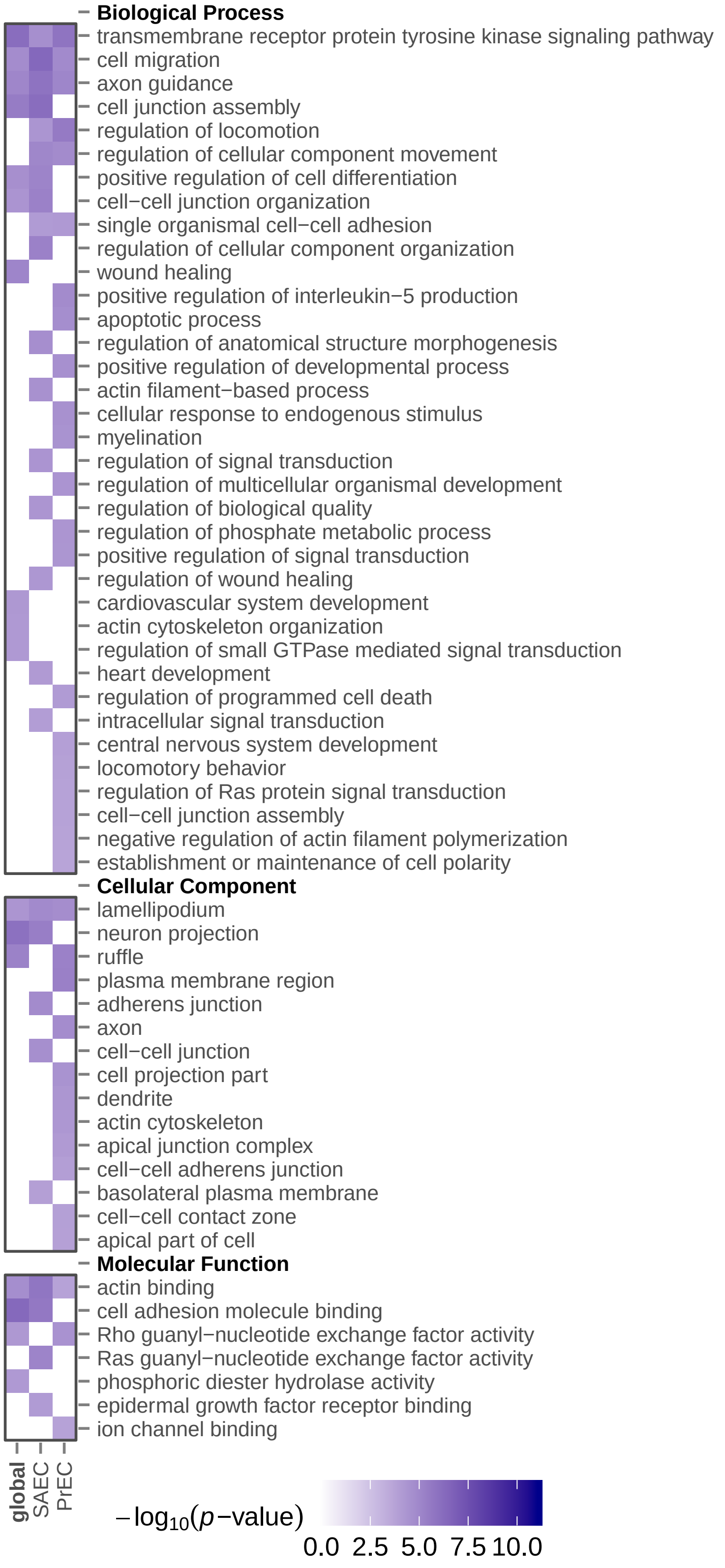

human ATF2;HMG−B M01010;M00172, Q6

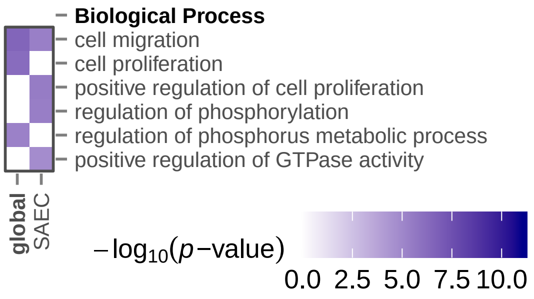

human ATF2;HMG−B M01010;M00801, Q0

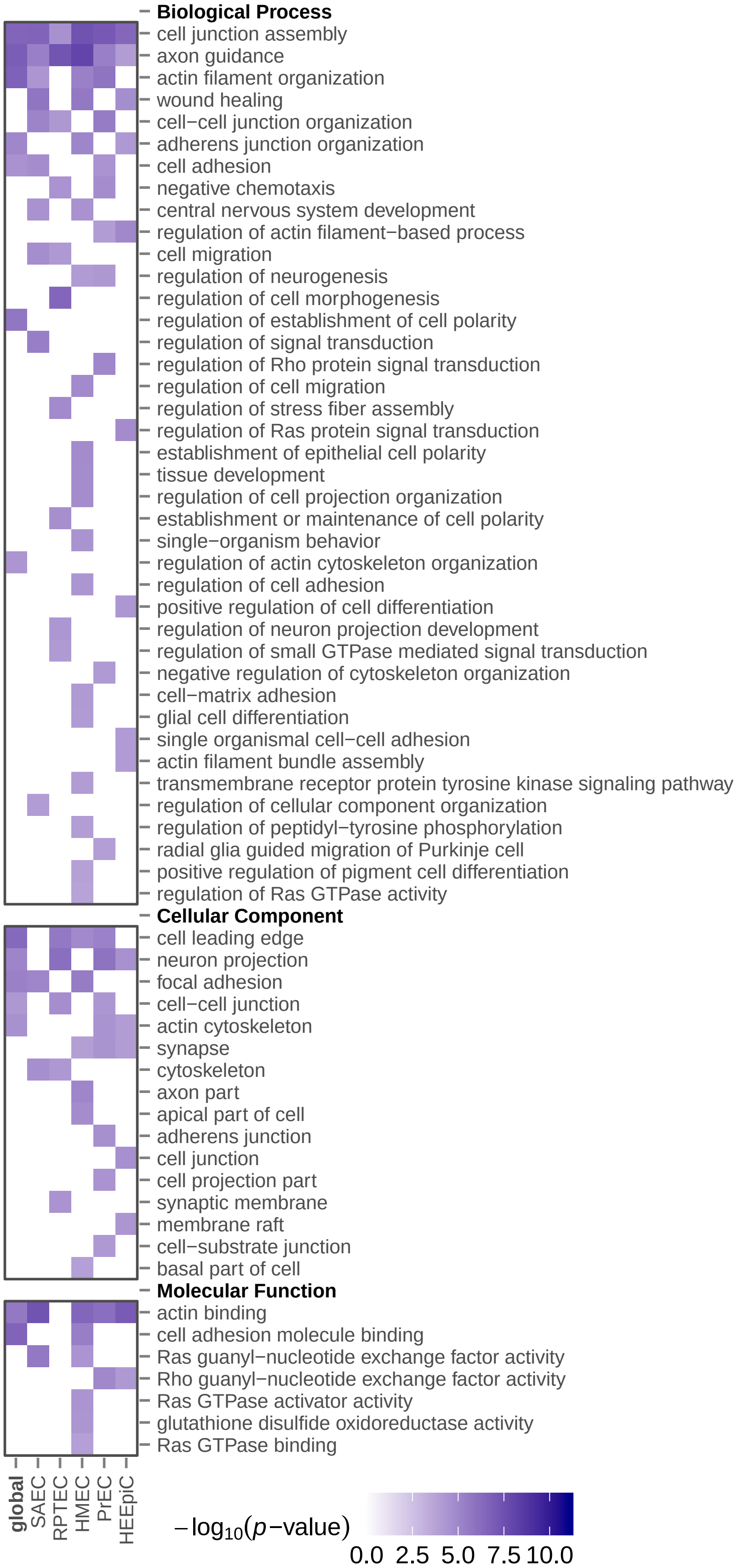

human ATF2;HMG−B M01010;M00801, Q6

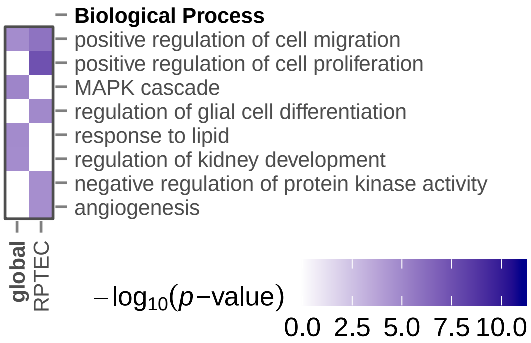

human HMG−A;ATF2 M00750;M00172, Q0

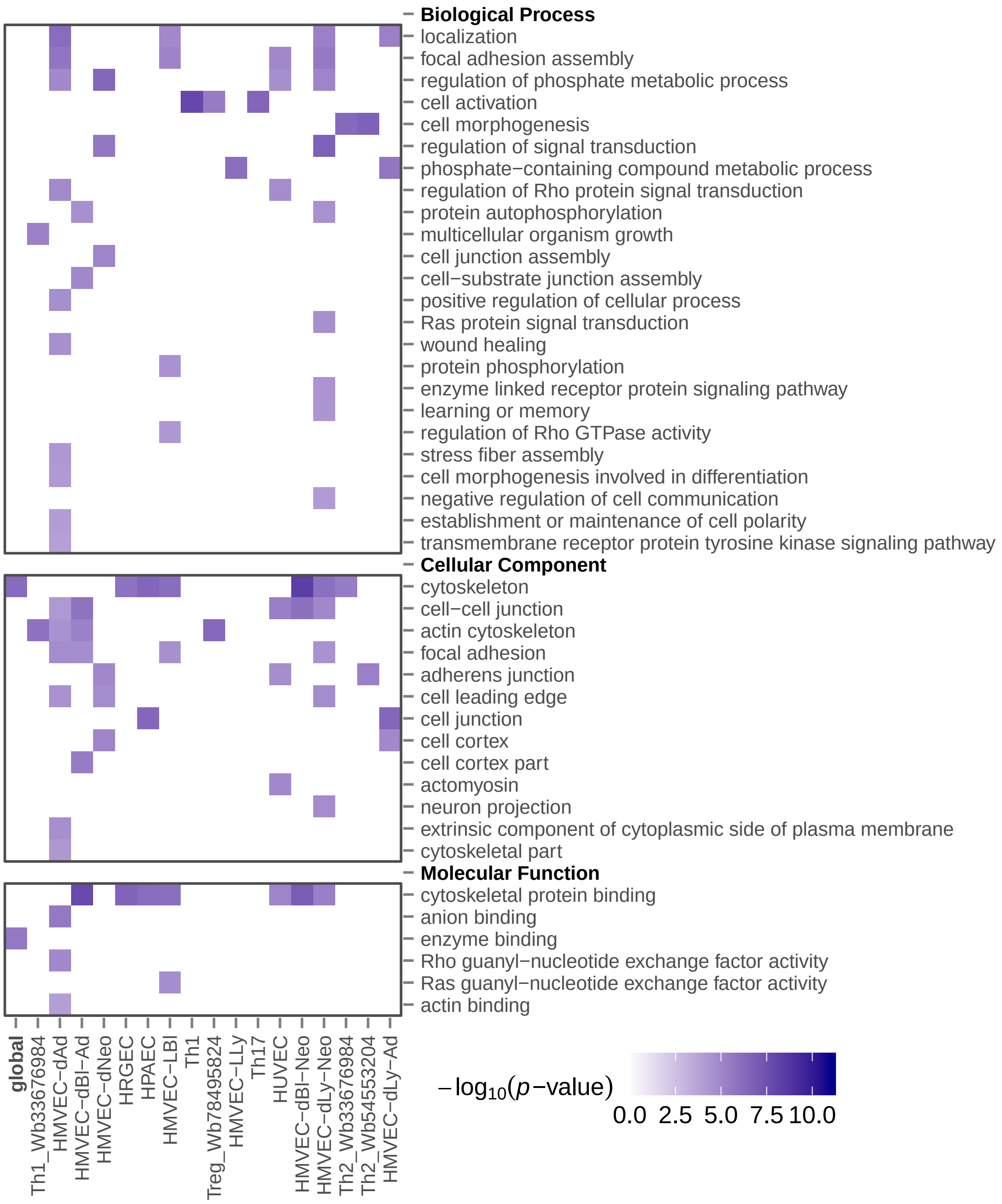

human HMG−A;ATF2 M00750;M00172, Q4

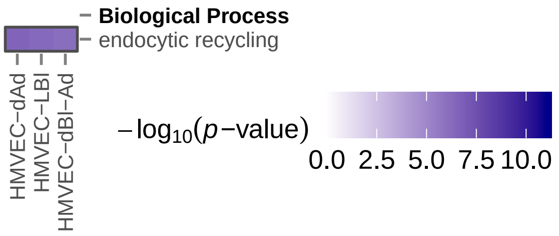

human HMG−A;ATF2 M00750;M00172, Q5

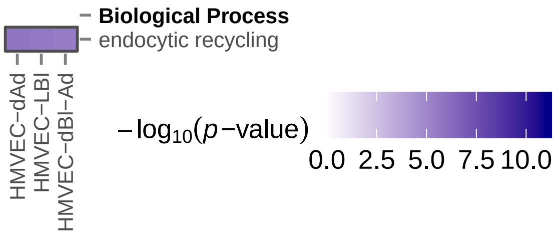

human HMG−A;ATF2 M00750;M00188, Q0

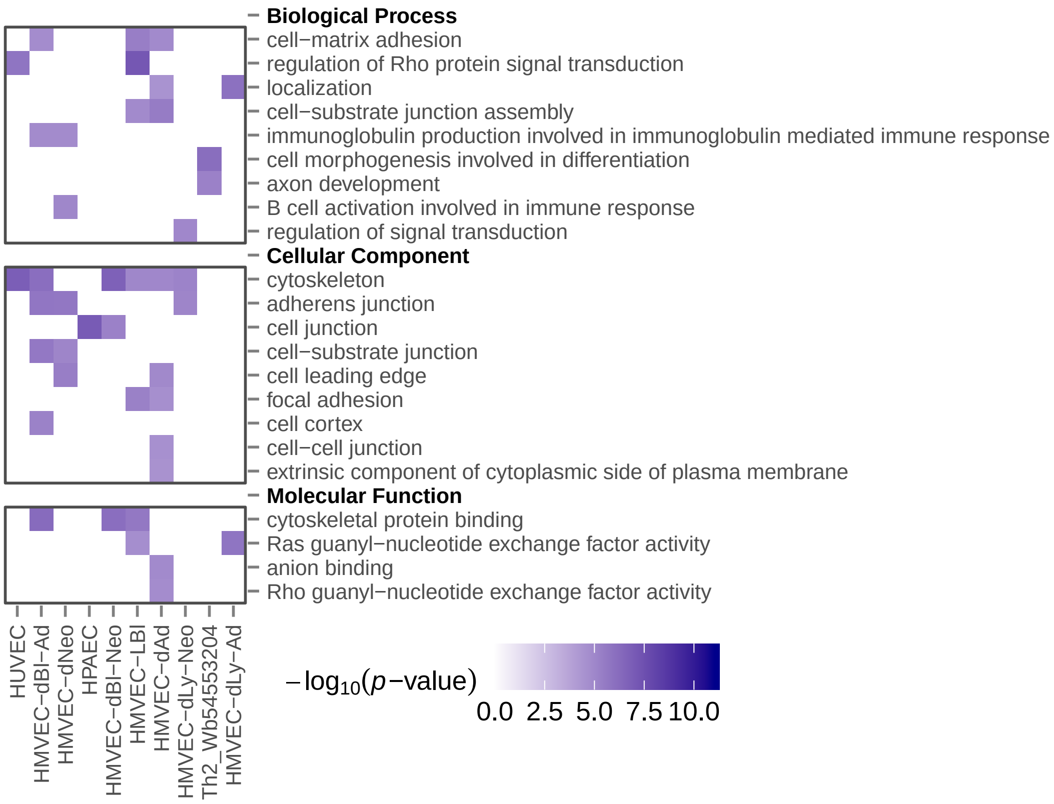

human HMG−A;ATF2 M00750;M00188, Q2

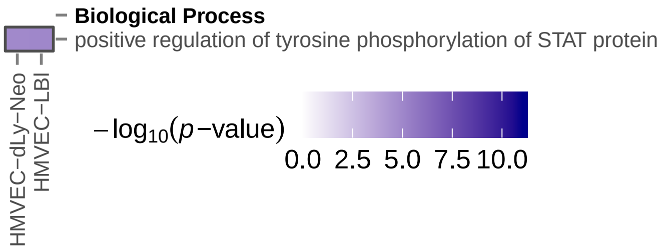

human HMG−A;ATF2 M00750;M00188, Q4

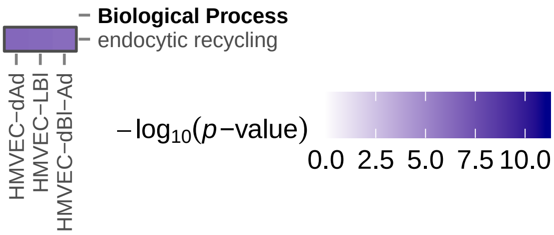

human HMG−A;ATF2 M00750;M00188, Q5

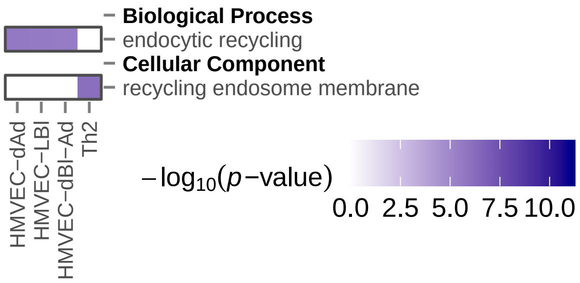

human HMG−A;ATF2 M00750;M00188, Q6

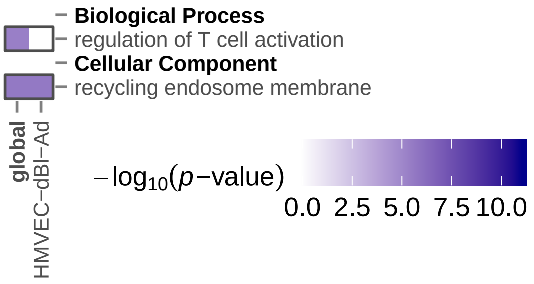

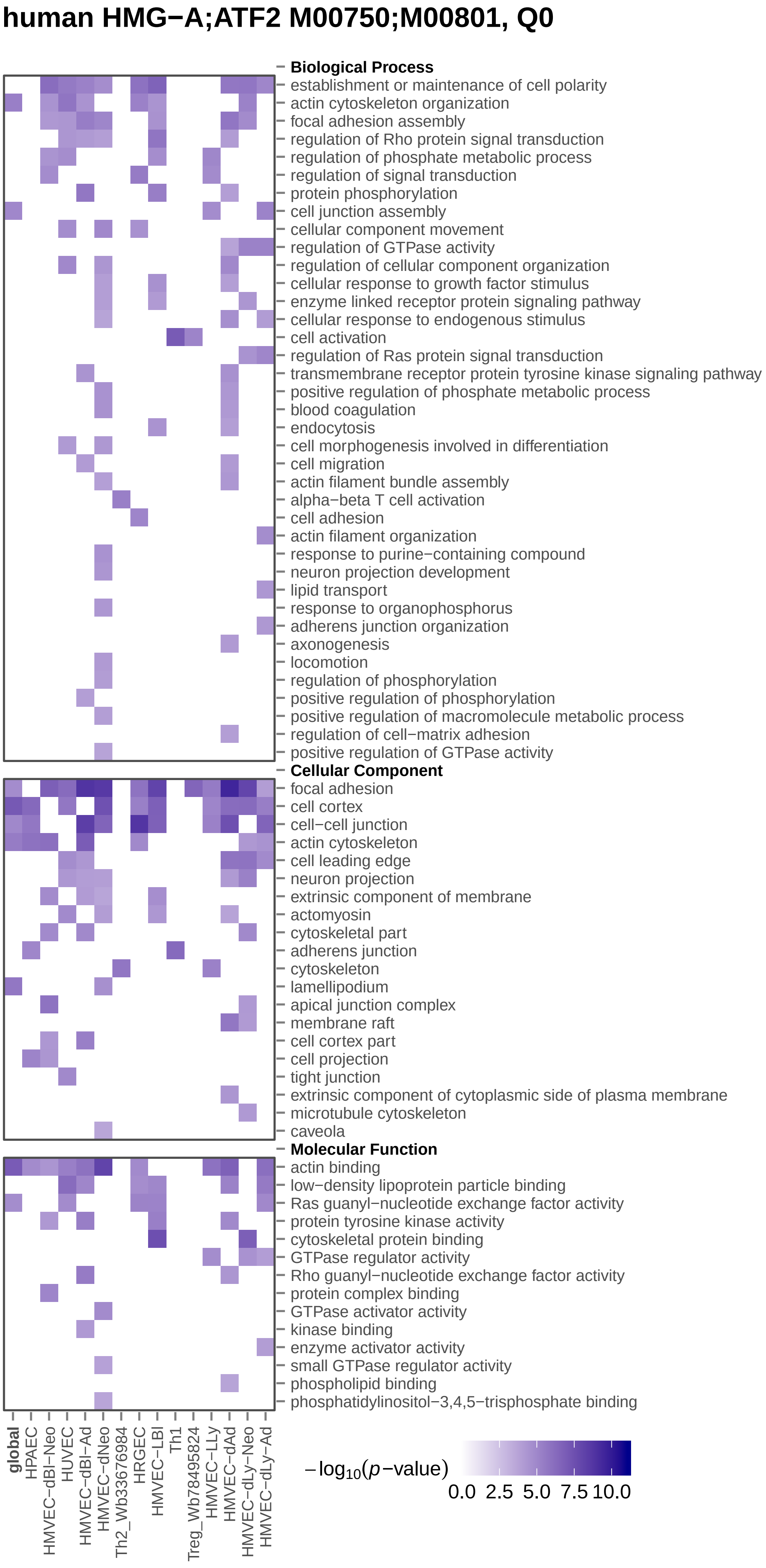
human HMG−A;ATF2 M00750;M00801, Q0
Biological Process
establishment or maintenance of cell polarity
actin cytoskeleton organization
focal adhesion assembly
regulation of Rho protein signal transduction
regulation of phosphate metabolic process
regulation of signal transduction
protein phosphorylation
cell junction assembly
cellular component movement
regulation of GTPase activity
regulation of cellular component organization
cellular response to growth factor stimulus
enzyme linked receptor protein signaling pathway
cellular response to endogenous stimulus
cell activation
regulation of Ras protein signal transduction
transmembrane receptor protein tyrosine kinase signaling pathway
positive regulation of phosphate metabolic process
blood coagulation
endocytosis
cell morphogenesis involved in differentiation
cell migration
actin filament bundle assembly
alpha−beta T cell activation
cell adhesion
actin filament organization
response to purine−containing compound
neuron projection development
lipid transport
response to organophosphorus
adherens junction organization
axonogenesis
locomotion
regulation of phosphorylation
positive regulation of phosphorylation
positive regulation of macromolecule metabolic process
regulation of cell−matrix adhesion
positive regulation of GTPase activity
Cellular Component
focal adhesion
cell cortex
cell−cell junction
actin cytoskeleton
cell leading edge
neuron projection
extrinsic component of membrane
actomyosin
cytoskeletal part
adherens junction
cytoskeleton
lamellipodium
apical junction complex
membrane raft
cell cortex part
cell projection
tight junction
extrinsic component of cytoplasmic side of plasma membrane
microtubule cytoskeleton
caveola
Molecular Function
actin binding
low−density lipoprotein particle binding
Ras guanyl−nucleotide exchange factor activity
protein tyrosine kinase activity
cytoskeletal protein binding
GTPase regulator activity
Rho guanyl−nucleotide exchange factor activity
protein complex binding
GTPase activator activity
kinase binding
enzyme activator activity
small GTPase regulator activity
phospholipid binding
phosphatidylinositol−3,4,5−trisphosphate binding
global
HPAEC
HMVEC−dBl−Neo
HUVEC
HMVEC−dBl−Ad
HMVEC−dNeo
Th2_Wb33676984
HRGEC
HMVEC−LBl
Th1
Treg_Wb78495824
HMVEC−LLy
HMVEC−dAd
HMVEC−dLy−Neo
HMVEC−dLy−Ad
−log10(p−value)
0.0 2.5 5.0 7.5 10.0

human HMG−A;ATF2 M00750;M00801, Q1

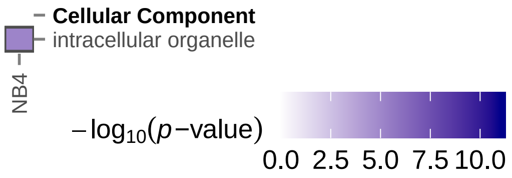

human HMG−A;ATF2 M00750;M00801, Q6

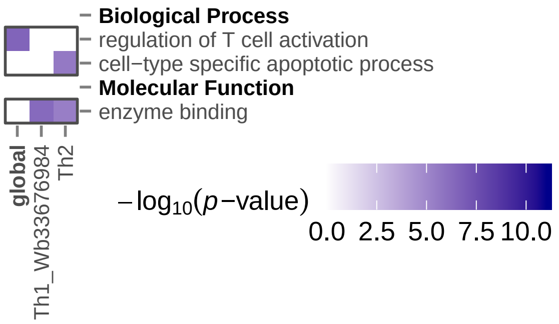

mouse HMG−A;ATF2 M00750;M00172, Q0

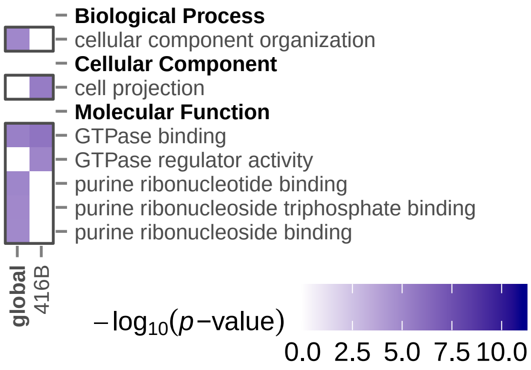

mouse HMG−A;ATF2 M00750;M00172, Q6

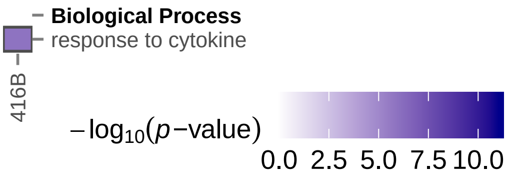

mouse HMG−A;ATF2 M00750;M00188, Q0

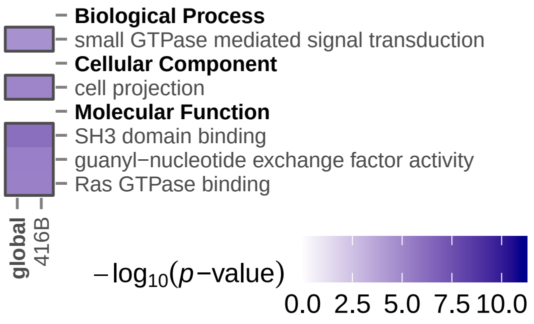

mouse HMG−A;ATF2 M00750;M00801, Q0

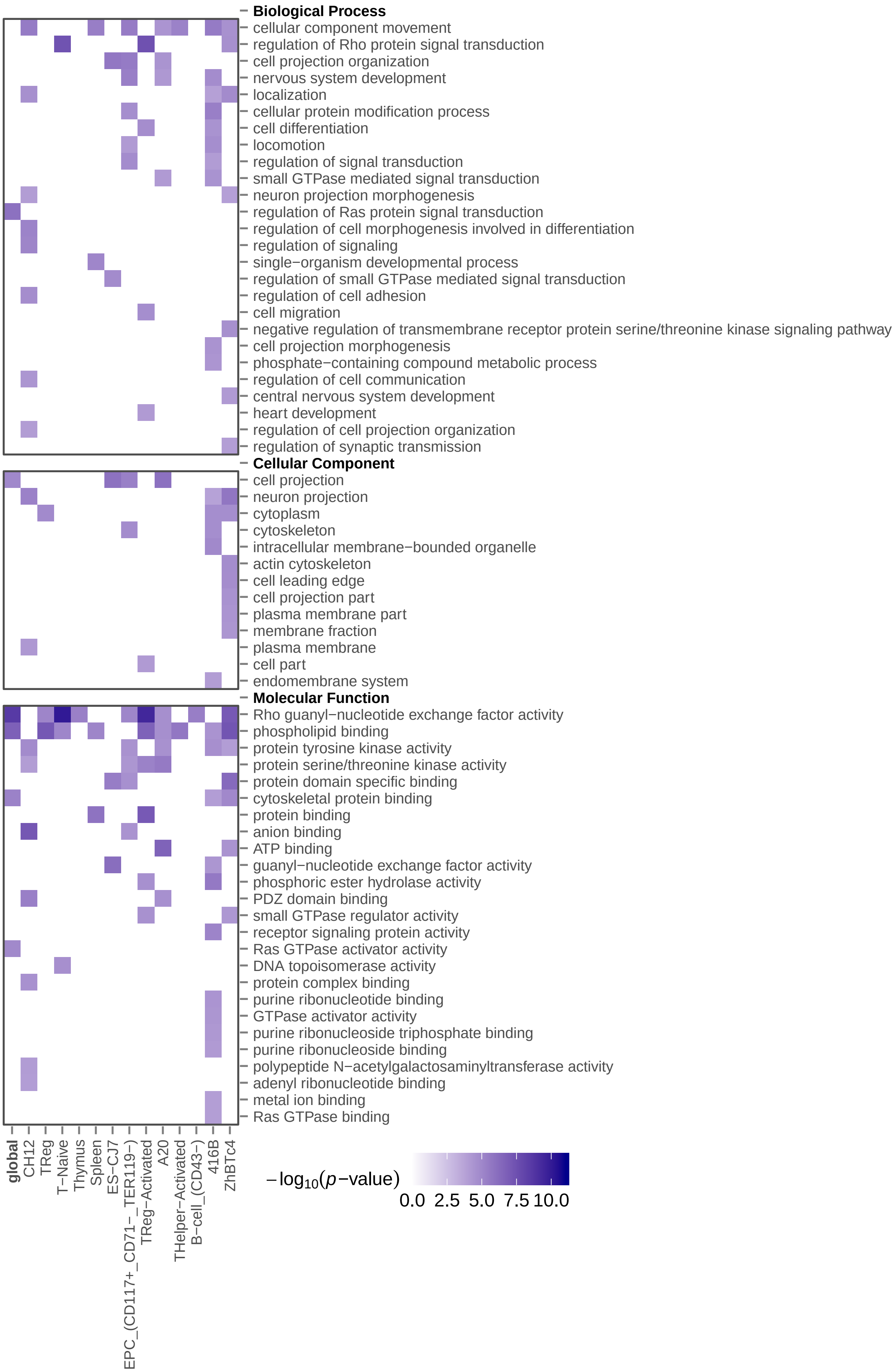

mouse HMG−A;ATF2 M00750;M00801, Q3

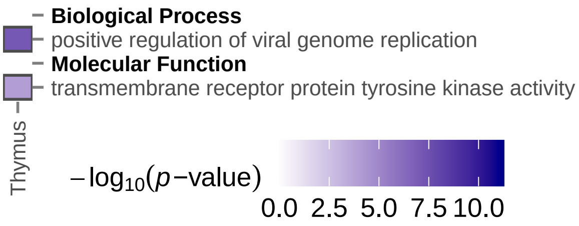

mouse HMG−A;ATF2 M00750;M00801, Q4

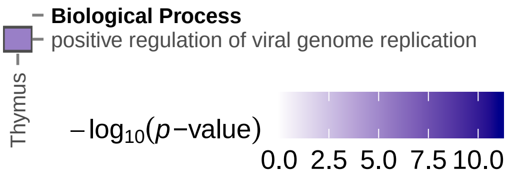

mouse IRF−A;IRF−B M00972;M01881, Q2

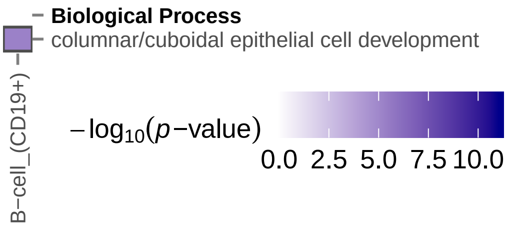

Supplement: Additional file 5 — Visualization of Gene Ontology enriched terms. Heatmap visualization of the Gene Ontology enriched terms with FDR <0.01 for predicted dimers. (PDF 60.1 kb) [file 12918_2016_258_MOESM5_ESM.pdf]
